# A statistical insight to exploration of medicinal wastewater as a source of thermostable lipase-producing microorganisms

**DOI:** 10.1371/journal.pone.0319023

**Published:** 2025-02-19

**Authors:** Sisir Rajak, Shaikh Rajesh Ali, Baidyanath Pal, Sibani Sen Chakraborty

**Affiliations:** 1 Department of Microbiology, Acharya Prafulla Chandra College, Kolkata, West Bengal, India; 2 Department of Microbiology, West Bengal State University, Kolkata, West Bengal, India; Pondicherry University, INDIA

## Abstract

Bacteria are ubiquitous and capable of thriving in diverse environments, including industrial effluents, which often present harsh physical and chemical conditions. These microorganisms produce various intracellular and extracellular biomolecules that enable adaptation, tolerance, and utilization of such extreme environments. Recognizing the growing industrial demand for thermostable lipases, this study focuses on the isolation, characterization, and optimization of lipase-producing bacteria from medicinal wastewater collected from a factory in North 24 Parganas, Kolkata, West Bengal, India. Nineteen lipase-producing bacterial isolates were obtained from nutrient agar plates and screened using tributyrin agar (TBA) plates. Extracellular lipolytic activity was confirmed via the cup-plate method with Tween 20/80 agar and methyl red as the indicator. The isolates were characterized morphologically and through biochemical tests. Extracellular lipase activity was quantified spectrophotometrically using para-nitrophenyl palmitate (pNPP) as a substrate in 50 mM Tris-HCl buffer, with absorbance measured at 410 nm after incubation at 65°C for 20 minutes to assess thermostability. Of the 19 isolates, 11 produced thermolabile lipases, while 8 exhibited thermostable lipase activity. Among these, three isolates (MWS14, MWS6, and MWS18) demonstrated high thermostable lipase production, with MWS18 being the most productive. Ribotyping and BLAST analysis revealed that these isolates shared 99% sequence similarity with Enterococcus, Bacillus, and Serratia species, respectively. Statistical analysis using the Kruskal-Wallis H-test confirmed significant differences in lipase production among the three groups of isolates. The study also predicts greater lipase production potential in Gram-negative bacterial strains compared to Gram-positive isolates. These findings highlight the industrial relevance of medicinal wastewater as a source of thermostable lipase-producing bacteria.

## Introduction

Industrial effluents, characterized by high levels of pollutants and extreme physicochemical conditions, are significant contributors to environmental contamination. However, these effluents create unique ecological niches that support the growth of microbial communities capable of adapting to and thriving in such harsh environments [1,2]. Microorganisms inhabiting these sites produce specialized enzymes that facilitate their survival and exploitation of these extreme conditions. Among these, microbial lipases have gained significant attention due to their versatility and diverse industrial applications [3,4].

Lipases (*triacylglycerol acylhydrolases*, EC 3.1.1.3) catalyze the hydrolysis of triglycerides into glycerol and free fatty acids at lipid-water interfaces. These enzymes are indispensable in several sectors, including food processing, detergents, textiles, pharmaceuticals, and bioenergy [5,6]. Additionally, they are instrumental in bioconversion processes and bioremediation due to their eco-friendly and efficient catalytic properties. Unlike chemical catalysts, lipases offer high specificity, lower energy requirements, and enhanced sustainability, making them integral to green industrial processes [7,8].

The increasing demand for thermostable lipases stems from their ability to maintain catalytic activity and stability under extreme conditions such as high temperatures, pH variations, and the presence of organic solvents [9,10]. These properties make thermostable lipases particularly valuable for industrial applications, including biodiesel production and polymer synthesis [11,12]. Advances in protein engineering and molecular biology have enabled the discovery and development of lipases with enhanced properties, including improved thermal stability and activity [13]. While thermophilic bacteria have been extensively explored for thermostable lipase production, mesophilic microorganisms remain underutilized, presenting an opportunity for identifying novel enzymes with unique features [14,15,16].

Microbial lipases, particularly bacterial enzymes, are predominantly extracellular and are influenced by environmental factors such as temperature, pH, carbon and nitrogen sources, and dissolved oxygen levels [17]. Bacteria, fungi, and yeast have been identified as potential sources of lipases, with bacteria demonstrating remarkable versatility and high production levels [18]. These enzymes have been isolated from diverse habitats, including industrial effluents, oil-contaminated soils, vegetable oil processing units, and dairy waste [5,19–22]. Industrial effluents, due to their complex chemical makeup and selective pressure on microbial populations, serve as rich reservoirs for isolating lipase-producing microorganisms [1,23–25].

While several studies have documented thermostable lipases from thermophilic bacteria, the exploration of mesophilic strains has been limited, presenting an opportunity for identifying novel enzymes with unique features [16,26]. Novel thermostable lipases with resistance to denaturation and sustained activity under elevated temperatures are essential for high-temperature industrial processes [22]. Furthermore, these enzymes have shown enhanced stability in the presence of solvents, detergents, and acidic or alkaline pH conditions, further expanding their industrial relevance [17,27,28].

This study investigates medicinal wastewater as a potential source for thermostable lipase-producing bacteria. Employing a combination of morphological, biochemical, and molecular techniques, it characterizes the isolates and evaluates their lipase production using statistical tools. These findings underscore the potential of medicinal effluents as an untapped resource for industrially significant enzymes.

## Materials & methods

### Isolation and screening of lipase-producing bacteria

Industrial wastewater samples were collected from a medicinal factory located in North 24 Parganas, Kolkata, West Bengal, India. One milliliter of the collected sample was diluted in 99 mL of 0.8% saline solution, and solid debris was precipitated by centrifugation at 100 rpm for 30 minutes at room temperature. The supernatant was serially diluted up to 10^ −8^, and 0.1 mL aliquots from the last three dilutions were plated onto nutrient agar plates. The plates were incubated at 37°C for 48 hours.

Individual bacterial colonies from the 10^ −7^ dilution were replica-plated onto Tributyrin Agar (TBA) medium containing 0.3% yeast extract (w/v), 0.5% peptone (w/v), 2% agar (w/v), and 1% tributyrin (v/v) at pH 7.0. Plates were incubated at 37°C for 48 hours, and lipase production was indicated by clear zones around colonies. Positive colonies were further confirmed by replating on fresh TBA medium [3].

To qualitatively and quantitatively assess lipolytic activity, positive isolates were inoculated into nutrient broth and incubated at 37°C with shaking at 150 rpm for 24 hours. The cultures were centrifuged at 8,000 rpm for 5 minutes to collect the supernatant. Lipase activity was analyzed on Tween 20 and Tween 80 agar plates containing 1% Tween 20 or Tween 80 and 0.02% methyl red indicator in 2% agar [2]. Zones of clearance were recorded as an indication of lipase activity.

### Morphological and biochemical characterization

For morphological studies, fresh cultures of the lipase-producing isolates were grown in nutrient broth at 37°C for 48 hours under shaking conditions. Gram staining was performed to determine the Gram reaction and morphological characteristics. Biochemical characterization was carried out using standard tests, including catalase activity, indole production, methyl red (MR) test, Voges-Proskauer (VP) test, and citrate utilization. These tests provided preliminary identification and characterization of the isolates [18].

### Screening for thermostable lipase production

To evaluate thermostability, the positive lipase-producing isolates were grown in nutrient broth for enzyme production. After 24 hours of incubation at 37°C with shaking, the cultures were centrifuged at 8,000 rpm for 8 minutes, and the supernatants were collected as crude enzyme preparations.

Lipase activity was assessed spectrophotometrically using *p*-nitrophenyl palmitate (*p*NPP) as a substrate [13,29]. A 20 mM *p*NPP stock solution was prepared in high-performance liquid chromatography (HPLC)-grade isopropanol. The reaction mixture consisted of 0.1 mL of 20 mM *p*NPP, 1.8 mL of 50 mM Tris-HCl buffer (pH 7.0), and 0.1 mL of crude enzyme. The mixture was incubated at 65°C for 20 minutes in a water bath. The reaction was terminated by adding an equal volume of ethanol-acetone mixture (1:1). Lipase activity was measured by detecting the release of *p*-nitrophenol (*p*NP) at 410 nm using a spectrophotometer.

A standard curve of *p*NP (6–27 µg/mL) was used to calculate enzyme activity. Lipase activity was expressed in international units (IU), where one unit is defined as the amount of enzyme that produces 1 µmol of *p*NP per minute under standard assay conditions. Thermostable lipase-producing isolates were selected for further study and preserved in 50% (v/v) glycerol stocks at −20°C [17].

### Ribotyping of potential thermostable lipase-producing bacteria

The genomic DNA of three selected thermostable lipase-producing isolates was extracted using a commercial DNA extraction kit (Eurofins, cat. no. 5224700305). The 16S rDNA region was amplified by polymerase chain reaction (PCR) using universal 16S-F and 16S-R primers. Amplification products were confirmed by electrophoresis on a 1% agarose gel. The PCR amplicons were purified to remove contaminants and sequenced.

Partial sequences of the 16S rDNA were analyzed using the BLAST tool (http://www.blast.ncbi.nlm.nih.gov) to identify the isolates based on maximum identity scores. The top ten matching sequences were aligned using ClustalW software. A phylogenetic tree was constructed using MEGA11 software to determine the evolutionary relationships of the isolates [9].

### Statistical analysis of lipase producers

Statistical analyses were performed using SPSS software (version 26). Data from the experiments were subjected to descriptive statistics and hypothesis testing, including proportion tests and the Kruskal-Wallis test. Additionally, classification and regression tree [CART] analysis was employed to classify the lipase-producing isolates based on their activity levels. All experiments were conducted in triplicates, and results were presented as mean ±  standard deviation [5].

## Results and discussion

### Isolation and screening of lipase-producing bacteria

**Fig 1:**
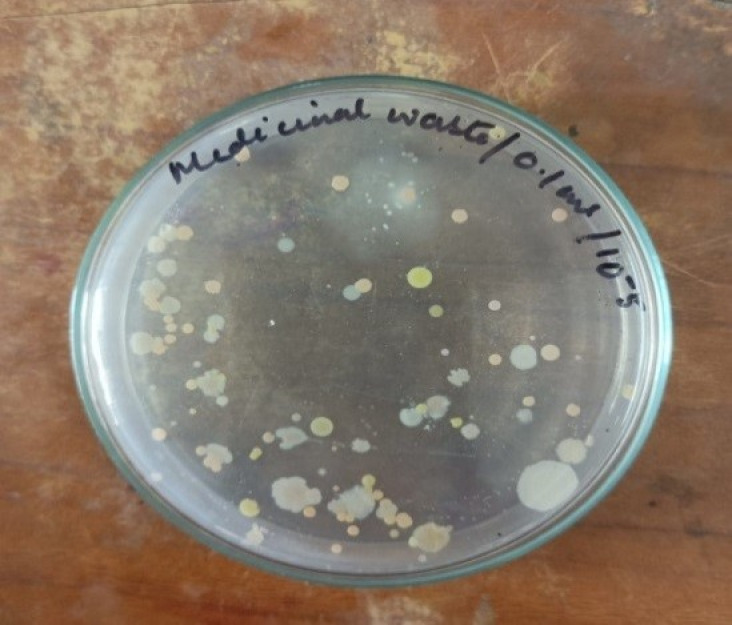
Viable count of bacteria in Nutrient agar media.

**Fig 2:**
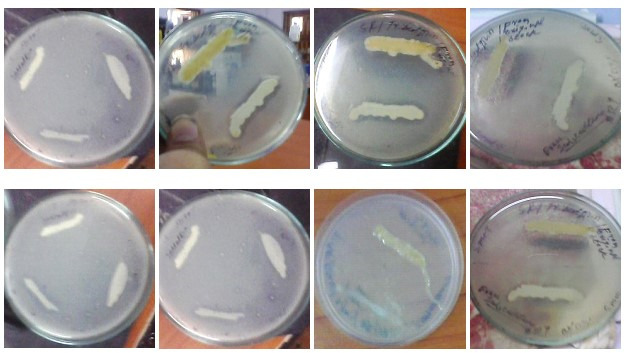
Lipolytic activity of few isolates in Tributyrin agar plate.

The viable count from the industrial wastewater sample was found to be 30 × 10^8^CFU/mL, indicating a high bacterial population in the medicinal waste environment (Fig 1). From these, 19 colonies demonstrated lipase-producing potential by forming clear halo zones on Tributyrin Agar (TBA) plates (Fig 2). Halo formation on TBA plates is a reliable preliminary indicator of lipase activity, as tributyrin is hydrolyzed by lipases to release free fatty acids, forming visible zones of clearance.

**Fig 3:**
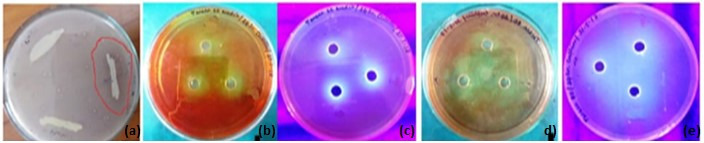
Lipolytic activity of bacteria in [a]TBA plate; [b]Tween-20 plate; [c]Tween-20 plate in UV; [d]Tween-80 plate; [e]Tween-80 plate in UV.

Further confirmation of lipolytic activity was performed using the cup-plate method on Tween 20/80 agar plates supplemented with a methyl red indicator. A halo under UV light confirmed the hydrolysis of Tween substrates, consistent with prior studies demonstrating this method’s sensitivity for detecting lipase activity (Fig 3) [2]. These results highlight the robustness of the screening approach in isolating active lipase producers from an industrial waste environment.

### Morphological and biochemical characteristics of isolated bacteria

**Fig 4:**
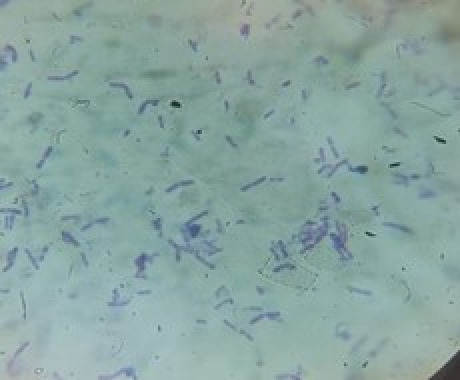
Gram positive isolate at 400X.

**Fig 5:**
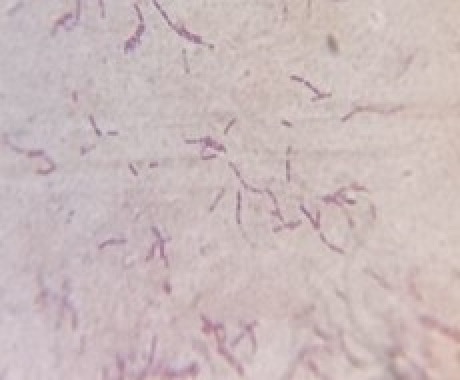
Gram negative isolate at 400X.

Morphological analysis revealed a diverse range of Gram-positive and Gram-negative bacteria among the 19 lipase producers. Specifically, strains MWS1, MWS6, MWS8, MWS11, and MWS14 were Gram-positive, while the remaining isolates were Gram-negative (Figs 4 and 5). The isolates exhibited varying shapes, with coccus forms observed in MWS1, MWS12, and MWS14, while others such as MWS3, MWS4, MWS6-9, MWS11, MWS13, and MWS16-19 were rod-shaped. A few isolates (MWS2, MWS10, and MWS15) displayed variable morphology (Table 1).

**Table 1:** Morphological and biochemical characterization of isolated lipase producers.

Sample Name	Indol	MR	VP	Citrate	Catalase	Gram Character	Shape	Lipase
MWS1	‒Ve	‒Ve	‒Ve	‒Ve	+Ve	+Ve	Coccus	+
MWS2	‒Ve	‒Ve	+Ve	‒Ve	‒Ve	‒Ve	Variable	+
MWS3	‒Ve	‒Ve	+Ve	‒Ve	‒Ve	‒Ve	Rod	+
MWS4	‒Ve	+Ve	‒Ve	‒Ve	‒Ve	‒Ve	Rod	+
MWS5	‒Ve	‒Ve	‒Ve	‒Ve	‒Ve	‒Ve	oval	+
MWS6	‒Ve	‒Ve	+Ve	+Ve	+Ve	+Ve	Rod	+
MWS7	‒Ve	‒Ve	‒Ve	‒Ve	‒Ve	‒Ve	Rod	+
MWS8	‒Ve	‒Ve	+Ve	+Ve	‒Ve	+Ve	Rod	+
MWS9	‒Ve	‒Ve	‒Ve	‒Ve	‒Ve	‒Ve	Rod	+
MWS10	‒Ve	‒Ve	‒Ve	‒Ve	‒Ve	‒Ve	Variable	+
MWS11	‒Ve	+Ve	+Ve	‒Ve/ + Ve	‒Ve/ + Ve	+Ve	Rod	+
MWS12	‒Ve	‒Ve	‒Ve	‒Ve	‒Ve	‒Ve	Coccus	+
MWS13	‒Ve	‒Ve	‒Ve	‒Ve	‒Ve	‒Ve	Rod	+
MWS14	‒Ve	+Ve	+Ve	‒Ve	‒Ve/ + Ve	+Ve	Coccus	+
MWS15	‒Ve	‒Ve	‒Ve	‒Ve	‒Ve	‒Ve	Variable	+
MWS16	‒Ve	+Ve	‒Ve	‒Ve	‒Ve	‒Ve	Rod	+
MWS17	‒Ve	+Ve	+Ve	‒Ve	‒Ve/ + Ve	‒Ve	Rod	+
MWS18	‒Ve	‒Ve	+Ve	+Ve	+Ve	‒Ve	Rod	+
MWS19	‒Ve	‒Ve	‒Ve	‒Ve	‒Ve	‒Ve	Rod	+

Biochemical characterization using tests such as catalase, indole production, methyl red, Voges-Proskauer, and citrate utilization further distinguished these isolates, suggesting their affiliation with distinct genera, as corroborated by earlier works on lipase-producing bacteria isolated from industrial wastes [18].

### Screening for thermostable lipase producers

**Fig 6:**
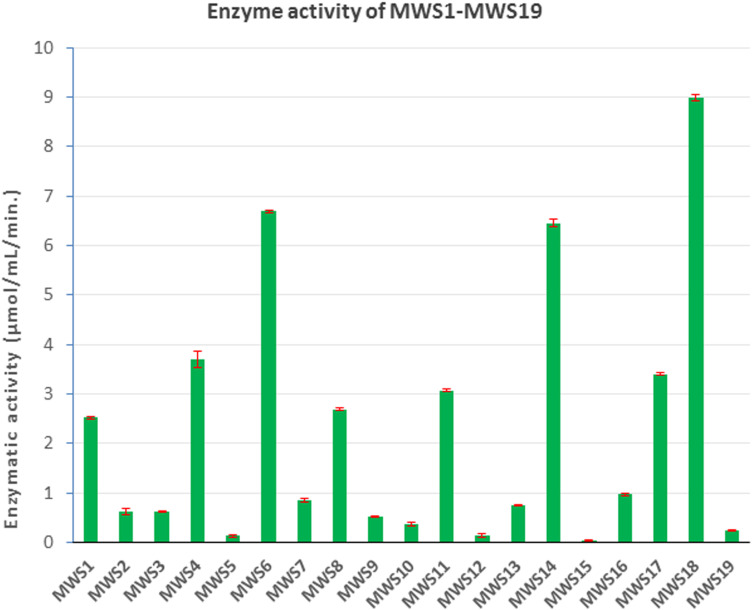
Crude enzyme activity of different lipase producers.

Among the 19 isolates, eight demonstrated the ability to produce thermostable lipases, with activity assessed spectrophotometrically using *p*-nitrophenyl palmitate (*p*NPP) as the substrate at 65°C. Of these, three strains ([MWS6, MWS14, and MWS18) showed the highest thermostable lipase production, with enzymatic activities of 6.69 ±  0.02, 6.45 ±  0.07, and 8.98 ±  0.07 µmol/mL/min, respectively (Fig 6, Table 2).

**Table 2:** Analyzing thermostability for different lipase producer.

Sample Name	Optical Density(OD) at 410nm	Enzyme Activity (µmol/mL/min)
MWS1	0.216 ± 0.002	2.527 ± 0.023
MWS2	0.056 ± 0.005	0.634 ± 0.062
MWS3	0.056 ± 0.001	0.6341 ± 0.011
MWS4	0.316 ± 0.014	3.710 ± 0.167
MWS5	0.014 ± 0.002	0.137 ± 0.023
MWS6	0.568 ± 0.002	6.69 ± 0.023
MWS7	0.075 ± 0.002	0.858 ± 0.031
MWS8	0.23 ± 0.002	2.692 ± 0.023
MWS9	0.047 ± 0.001	0.527 ± 0.011
MWS10	0.034 ± 0.002	0.373 ± 0.031
MWS11	0.262 ± 0.002	3.0711 ± 0.023
MWS12	0.015 ± 0.003	0.149 ± 0.042
MWS13	0.066 ± 0.002	0.752 ± 0.023
MWS14	0.548 ± 0.006	6.454 ± 0.073
MWS15	0.005 ± 0.001	0.030 ± 0.011
MWS16	0.084 ± 0.001	0.969 ± 0.013
MWS17	0.29 ± 0.002	3.402 ± 0.031
MWS18	0.762 ± 0.006	8.986 ± 0.071
MWS19	0.023 ± 0.001	0.243 ± 0.020

Thermostable lipases are of significant interest for industrial applications due to their ability to retain activity under high temperatures, which is critical for processes such as biodiesel production and waste management [13]. These findings align with previous reports where thermostable lipases were preferentially isolated from high-temperature environments.

### Identification of enterococcus, bacillus, and Serratia using 16S rDNA analysis as novel thermostable lipase producer

**Fig 7:**
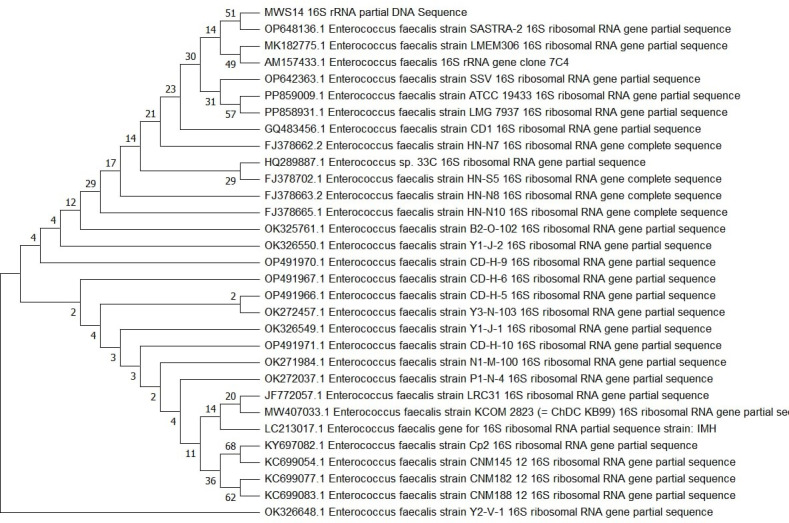
Phylogenetic analysis based on 16SrDNA for strain MWS14.

**Fig 8:**
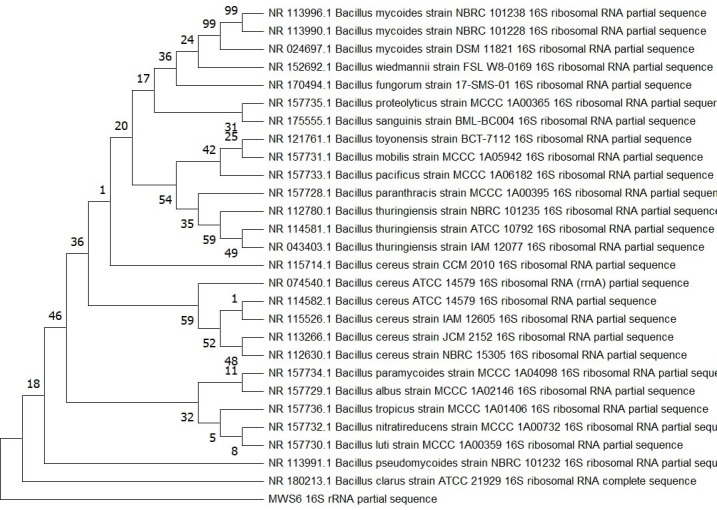
Phylogenetic analysis based on 16SrDNA for strain MWS6.

**Fig 9:**
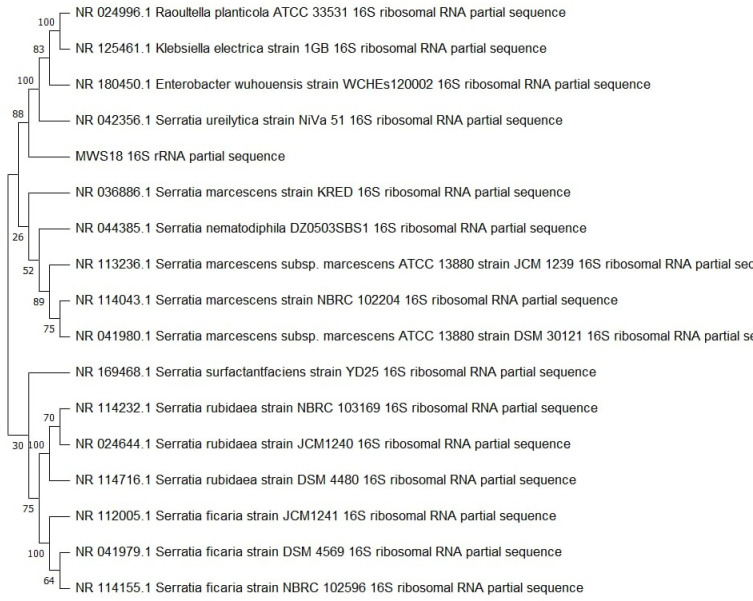
Phylogenetic analysis based on 16SrDNA for strain MWS18.

The three potential thermostable lipase producers were identified through 16S rDNA sequencing. BLAST analysis revealed 99% similarity with known species, assigning MWS14, MWS6, and MWS18 to the genera *Enterococcus*, *Bacillus*, and *Serratia*, respectively. Phylogenetic trees constructed for these isolates confirmed their genetic affiliations and evolutionary relationships (Figs 7–9).

The identification of *Serratia sp.*, *Bacillus sp.*, and *Enterococcus sp.* as novel thermostable lipase producers is notable, as these genera have been reported in previous studies for their industrially relevant enzyme profiles [30,31]. These results further expand the understanding of their enzymatic potential in diverse environments.

### Statistical analysis of lipase producers

A one-sample proportion test was conducted to evaluate the statistical significance of the observed sample proportion of lipase producers (test summary in supplementary file). The sample proportion (p = 0.633) was compared against the hypothesized population proportion [P = 0.5]. The Z-test yielded a test statistic of 1.46 with a p-value of 0.144, indicating no significant difference between the sample and population proportions (p >  0.05). Thus, the null hypothesis was accepted, affirming the unbiased nature of the sample proportion (CI_95_: 0.4609–0.8058).

Descriptive statistics revealed that the category of high thermostable lipase producers had the highest mean and standard deviation of activity values, indicating greater variability in this group compared to thermolabile producers, which formed a more homogeneous group. These observations align with the notion that thermostable lipase production is influenced by genetic and environmental factors, leading to heterogeneous outcomes within strains [9].

**Fig 10:**
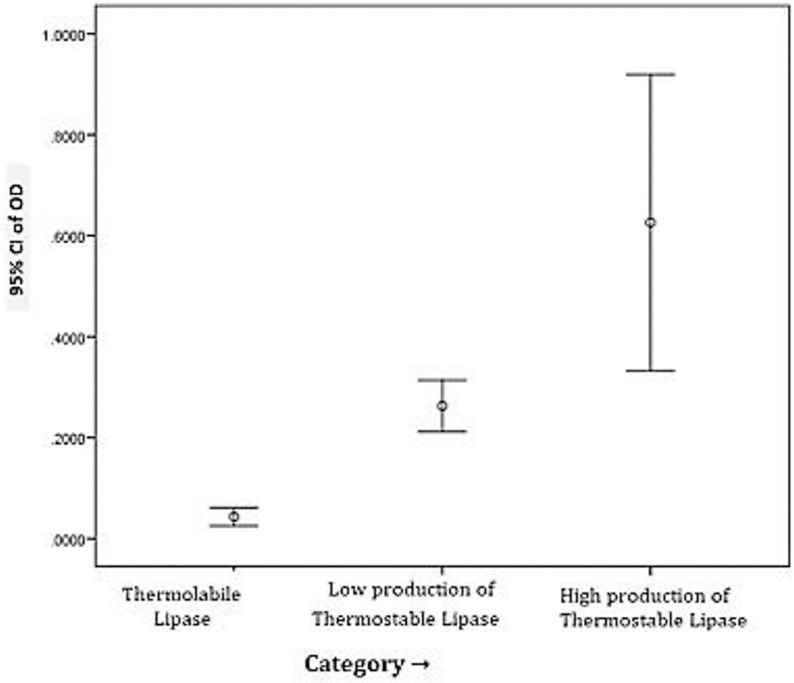
Significant mean difference among three categories.

The Kruskal-Wallis test further supported these findings (supplementary file), showing statistically significant differences in optical density [OD] values across the three categories of lipase producers (χ² =  14.16, p =  0.001). High thermostable lipase producers had the highest mean ranks, underscoring their superior activity levels (Fig 10).

### CART analysis for predictive modelling

**Fig 11:**
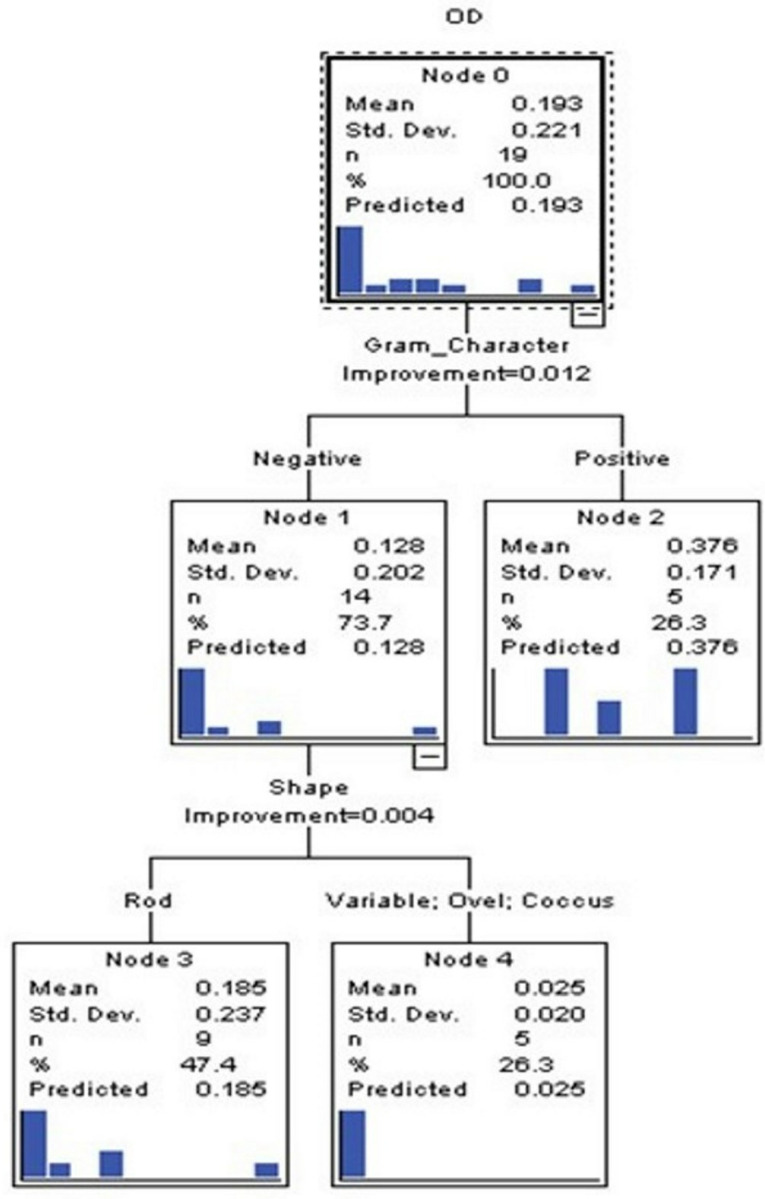
Categorical Regression tree.

**Fig 12:**
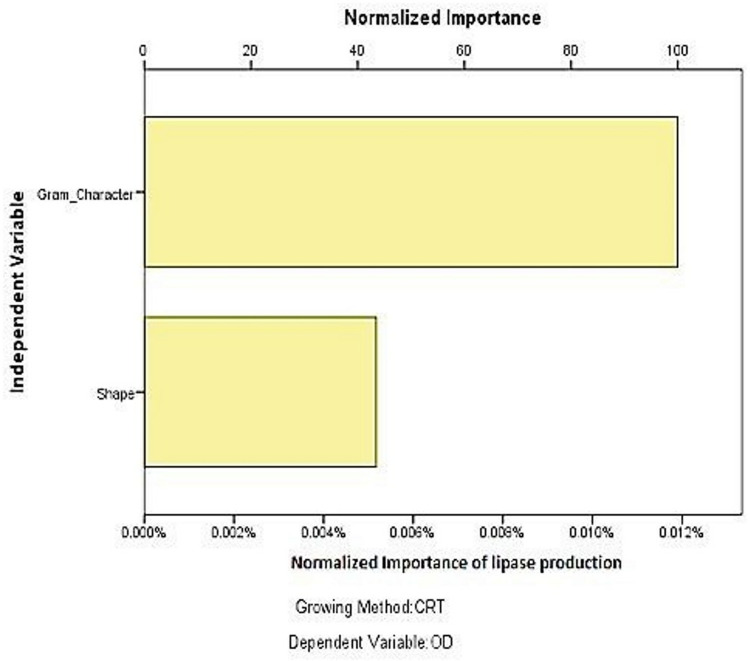
CART prediction for lipase production among the morphological variable.

Classification and Regression Tree (CART) analysis (Fig 11) identified Gram character as a key predictor of lipase production potential (Fig 12). The decision tree showed that Gram-negative bacteria were more likely to produce lipase, with an accuracy of 73.7%. Further bifurcation by bacterial shape revealed that rod-shaped Gram-negative bacteria were particularly associated with higher lipase activity. This result is consistent with reports highlighting the prevalence of Gram-negative lipase producers in environmental samples due to their adaptive enzymatic systems [2].

In summary, this study identified three potent thermostable lipase-producing strains with potential industrial applications. Statistical analyses and predictive modelling further underscored the influence of bacterial morphology and Gram character on lipase production. These findings contribute to the growing body of knowledge on microbial lipase diversity and functionality, particularly in high-temperature industrial processes.

## Conclusion

This study successfully isolated 19 lipase-producing bacterial colonies from industrial wastewater, with a viable count of 30 ×  10⁸ CFU/mL on nutrient agar. Initial screening on Tributyrin Agar (TBA) plates identified potential lipase producers through halo zone formation. Of these, 11 isolates produced thermolabile lipase, while 8 exhibited thermostable lipase activity. Notably, three isolates—MWS 6, MWS 14, and MWS 18—demonstrated high thermostable lipase production with crude enzyme activities of 6.69 ±  0.02, 6.45 ±  0.07, and 8.98 ±  0.07 µmol/mL/min, respectively.

BLAST analysis confirmed that MWS14, MWS6, and MWS18 shared 99% sequence similarity with species from the genera Enterococcus, Bacillus, and Serratia, respectively. Among these, MWS18 (Serratia sp.) showed the highest production of thermostable lipase, underscoring its industrial potential.

Statistical evaluations revealed significant differences in lipase activity among thermolabile and thermostable producers. Additionally, Classification and Regression Tree (CART) analysis highlighted the importance of Gram character over bacterial shape in predicting lipase production potential. These findings emphasize the suitability of three potential isolates from the genera Enterococcus, Bacillus, and Serratia for applications in industrial processes requiring thermostable enzymes, contributing to the broader understanding of microbial lipase diversity and its biotechnological applications.

## Supporting information

S1 File Bar diagram + pNP standard. (XLSX)

S2 File Specify hypotheses. (DOCX)

S3 File Descriptive Statistics. (DOCX)

S4 File Kruskal Wallis test. (DOCX)

## References

[1] Das K, Sarkar S, Banerjee S. Microbial diversity in industrial effluents: a treasure trove for novel enzyme discovery. Environ Microbiol Rep. 2022;14(5):662–75.

[2] Ahmed R, Sen S. Role of microbial enzymes in mitigating industrial pollution. Environ Biotechnol Rep. 2023;8(2):122–34.

[3] Sharma M, Gupta R, Mehta P. Industrial effluents as a source of enzyme-producing microbes: a sustainable approach. J Clean Prod. 2023;398:136965.

[4] Singh G, Pandey S. Bacterial lipases and their industrial applications: an emerging trend. Bioresour Technol Rep. 2022;18:101067.

[5] Patel A, Verma S, Shah K. Exploring microbial lipases for sustainable bio-industrial applications. Renew Energy. 2023;189:220–30.

[6] Huang Y, Ma L, Zhou Y. Recent advancements in microbial lipase technology and its industrial applications. Microb Cell Fact. 2021;20:174.34488765

[7] Karadzic I, Masui A, Cutfield JF. Lipases from extremophilic microorganisms and their industrial applications. Appl Microbiol Biotechnol. 2020;104:1339–54.

[8] Holkar CR, Jadhav AJ, Pinjari DV. Industrial effluent treatment using microbial lipases: current developments and future perspectives. Biochem Eng J. 2020;164:107775.

[9] Zhang X, Liu Q, Wei J. Applications of thermostable lipases in biofuels and bioplastics. Renew Sustain Energy Rev. 2022;156:111880.

[10] Sundar K, Rai A. Applications of thermostable enzymes in biofuel and bioethanol production. J Clean Prod. 2023;401:126444.

[11] Gupta R, Gupta N, Rathi P. Bacterial lipases: an overview of production, purification, and biochemical properties. Microb Biotechnol. 2021;64(6):763–81. doi: 10.1007/s00253-004-1568-8 14966663

[12] Pandey A, Benjamin S, Soccol CR. Advances in microbial lipase technology: current status and future directions. Renew Energy. 2021;164:1342–56.

[13] Kumar P, Sharma N, Singh D. Protein engineering approaches for enhancing lipase thermostability: recent trends and future perspectives. J Mol Catal B Enzym. 2021;172:102347.

[14] Gao X, Zhi Y, Zhang X. Mechanistic insights into thermostable lipases: a review. Chem Biol Drug Des. 2021;97(6):1234–43.

[15] Kumar A, Thakur V. Industrial exploitation of thermostable enzymes: advances and challenges. Biotechnol Rep. 2022;35:e00688.

[16] Rai R, Singh K, Chauhan D. Novel mesophilic microbial lipases: a promising alternative for industrial processes. Front Bioeng Biotechnol. 2023;11:102391.

[17] Mondal R, Sarkar A, Saha B. Stability and activity of microbial lipases under industrial conditions: a comparative analysis. Enzyme Microb Technol. 2023;159:110034.

[18] Ghosh T, Mukherjee A, Chatterjee D. Bacterial lipases: production, characterization, and industrial applications. Biocatal Biotransform. 2023;41(2):165–78.

[19] Sirisha E, Rajasekar N, Narasu ML. Isolation and optimization of lipase-producing bacteria from oil-contaminated soils. Adv Biol Res. 2010;4(5):249–52.

[20] Golani M, Hajela K, Pandey GP. Screening, identification, characterization, and production of bacterial lipase from oil-spilled soil. Int J Curr Microbiol Appl Sci. 2016;5(3):745–63. doi: 10.20546/ijcmas.2016.503.087

[21] Rakshit SK, Guha A. Bacterial lipase production: optimization and scale-up for industrial use. J Bioprocess Eng. 2021;25(4):92–101.

[22] Roy P, Sengupta S, Paul A. Enhancing thermostable lipase production from mesophilic bacteria: challenges and opportunities. Appl Microbiol Biotechnol. 2023;107(2):411–23.

[23] Aktar L, Khan FI, Islam T, Mitra S, Saha ML. Isolation and characterization of indigenous lipase-producing bacteria from lipid-rich environments. Plant Tissue Culture Biotechnol. 2016;26(2):243–53. doi: 10.3329/ptcb.v26i2.30574

[24] Ilesanmi OI, Adekunle AE, Omolaiye JA, Olorode EM, Ogunkanmi AL. Isolation, optimization, and molecular characterization of lipase-producing bacteria from contaminated soil. Sci Afr. 2020;8:e00279. doi: 10.1016/j.sciaf.2020.e00279

[25] Fang W, Zhou C. Insights into the catalytic efficiency of thermostable microbial enzymes. Front Microbiol. 2023;14:1278.

[26] El-Batal AI, Karem HH. Novel thermostable lipases from bacterial sources for industrial purposes. Bioorg Chem. 2020;114:104846.

[27] Ahmed S, Gupta P, Singh R. Advances in microbial lipase production for industrial applications. Biotechnol Adv. 2022;48:107822.

[28] Saharan S, Kaushik S. Microbial lipases: a green alternative for biocatalysis. J Biocatal. 2021;9(2):89–96.

[29] Ali SR, Sultana SS, Rajak S, Tribedi P, Sen Chakraborty S. Serratia sp. scl1: isolation of a novel thermostable lipase-producing microorganism with industrial importance. Antonie Van Leeuwenhoek. 2022;115:1335–48. 36127621 10.1007/s10482-022-01776-y

[30] García-Silvera EE, Martínez-Morales F, Bertrand B, Morales-Guzmán D, Rosas-Galván NS, León-Rodríguez R, et al. Production and application of a thermostable lipase from *Serratia marcescens* in detergent formulation and biodiesel production. Biotechnol Appl Biochem. 2018;65(2):156–72. doi: 10.1002/bab.1565 28444972

[31] Patel P, Desai P. Isolation, identification, and production of lipase-producing bacteria from oil-contaminated soil. BMR Microbiol. 2018;4(1):1–7.

