# Supplementary material for: A statistical insight to exploration of medicinal wastewater as a source of thermostable lipase-producing microorganisms
Source: PLoS One. 2025 Feb 19;20(2):e0319023. doi: 10.1371/journal.pone.0319023 (PMC11838911; doi:10.1371/journal.pone.0319023)
Supplement: S2 File — (DOCX) [file pone.0319023.s002.docx]

| **Specify hypotheses:** |
| --- |

| Null hypothesis H0:P=p [P= population proportion and p = sample proportion of lipase positive] |
| --- |
| \| Alternative hypothesis \| Ha: P≠ p \| \| --- \| --- \| |

| **Test summary** | |
| --- | --- |
| Null hypothesis | H0:P=0.5 |
| Alternative hypothesis | Ha: P≠ 0.5 |
| Type I error rate | α=0.05 |
| Sample size | N=30, n= lipase positive [event of interest] = 19 |
| Sample proportion | 𝑝=0.6333 |
| Sample standard error | Sp=0.091287 |
| Test statistic | z=1.460593 |
| Prob. value | p=0.144127 |
| Decision | Do not reject the null hypothesis |
| 95 % Confidence interval | CI_95_=[0.460855, 0.805812] |
