# Supplementary material for: A statistical insight to exploration of medicinal wastewater as a source of thermostable lipase-producing microorganisms
Source: PLoS One. 2025 Feb 19;20(2):e0319023. doi: 10.1371/journal.pone.0319023 (PMC11838911; doi:10.1371/journal.pone.0319023)
Supplement: S3 File — (DOCX) [file pone.0319023.s003.docx]

**Descriptive Statistics based on optical density of lipase producers**

| Sample types | N | Mean | Std. Deviation | 95% Confidence Interval for Mean | | Minimum  Absorbance [O.D.]at 410nm | Maximum absorbance [O.D.] at 410nm |
| --- | --- | --- | --- | --- | --- | --- | --- |
|  |  |  |  | Lower Bound | Upper Bound |  |  |
| Thermolabile Lipase | 11 | 0.043 | 0.027 | 0.025 | 0.061 | 0.005 | 0.085 |
| Low production of Thermostable lipase | 5 | 0.263 | 0.041 | 0.211 | 0.314 | 0.216 | 0.316 |
| High production of Thermostable lipase | 3 | 0.626 | 0.118 | 0.332 | 0.920 | 0.548 | 0.762 |
| **Total** | **19** | **0.193** | **0.221** | **0.087** | **0.299** | **0.005** | **0.762** |
