# Supplementary figures and images for: A statistical insight to exploration of medicinal wastewater as a source of thermostable lipase-producing microorganisms
Source: PLoS One. 2025 Feb 19;20(2):e0319023. doi: 10.1371/journal.pone.0319023 (PMC11838911; doi:10.1371/journal.pone.0319023)

**Kruskal Wallis test [H-Test]**

**
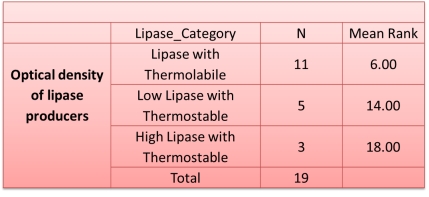
**

| Test Statisticsa,b | |
| --- | --- |
|  | OD |
| Chi-Square | 14.160 |
| df | 2 |
| Asymp. Sig.[ *p* ] | .001 |


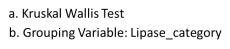

Supplement: S4 File — (DOCX) [file pone.0319023.s004.docx]
